# Effect of Methyl Jasmonate on the Terpene Trilactones, Flavonoids, and Phenolic Acids in *Ginkgo biloba* L. Leaves: Relevance to Leaf Senescence

**DOI:** 10.3390/molecules26154682

**Published:** 2021-08-02

**Authors:** Marcin Horbowicz, Wiesław Wiczkowski, Justyna Góraj-Koniarska, Kensuke Miyamoto, Junichi Ueda, Marian Saniewski

**Affiliations:** 1 Department of Plant Physiology, Genetics and Biotechnology, University of Warmia and Mazury, Oczapowskiego 1a, 10-719 Olsztyn, Poland; 2 Department of Chemistry and Biodynamics of Food, Institute of Animal Reproduction and Food Research of the Polish Academy of Sciences, Tuwima 10, 10-748 Olsztyn, Poland; 3 Research Institute of Horticulture, Konstytucji 3 Maja 1/3, 96-100 Skierniewice, Poland; justyna.goraj@inhort.pl (J.G.-K.); Marian.Saniewski@inhort.pl (M.S.); 4 Faculty of Liberal Arts and Sciences, Osaka Prefecture University, 1-1 Gakuen-cho, Naka-ku, Sakai, Osaka 599-8531, Japan; miyamoto@las.osakafu-u.ac.jp; 5 Department of Biological Science, Graduate School of Science, Osaka Prefecture University, 1-1 Gakuen-cho, Naka-ku, Sakai, Osaka 599-8531, Japan; ueda@b.s.osakafu-u.ac.jp

**Keywords:** *Ginkgo biloba*, ginkgolides, bilobalide, flavonoids, phenolic acids, leaf senescence, methyl jasmonate

## Abstract

The present study compared the effects of natural senescence and methyl jasmonate (JA-Me) treatment on the levels of terpene trilactones (TTLs; ginkgolides and bilobalide), phenolic acids, and flavonoids in the primary organs of *Ginkgo biloba* leaves, leaf blades, and petioles. Levels of the major TTLs, ginkgolides B and C, were significantly higher in the leaf blades of naturally senesced yellow leaves harvested on 20 October compared with green leaves harvested on 9 September. In petioles, a similar effect was found, although the levels of these compounds were almost half as high. These facts indicate the importance of the senescence process on TTL accumulation. Some flavonoids and phenolic acids also showed changes in content related to maturation or senescence. Generally, the application of JA-Me slightly but substantially increased the levels of TTLs in leaf blades irrespective of the difference in its application side on the leaves. Of the flavonoids analyzed, levels of quercetin, rutin, quercetin-4-glucoside, apigenin, and luteolin were dependent on the JA-Me application site, whereas levels of (+) catechin and (−) epicatechin were not. Application of JA-Me increased ferulic acid and *p*-coumaric acid esters in the petiole but decreased the levels of these compounds in the leaf blade. The content of *p*-coumaric acid glycosides and caffeic acid esters was only slightly modified by JA-Me. In general, JA-Me application affected leaf senescence by modifying the accumulation of ginkogolides, flavonoids, and phenolic acids. These effects were also found to be different in leaf blades and petioles. Based on JA-Me- and aging-related metabolic changes in endogenous levels of the secondary metabolites in *G. biloba* leaves, we discussed the results of study in the context of basic research and possible practical application.

## 1. Introduction

*Ginkgo biloba* (L.) is the only surviving member of the *Ginkgoaceae* family, one of the most ancient living gymnosperms, and is planted worldwide as an ornamental tree for its resistance to urban conditions [1]. *G. biloba* is also one of the most commercialized medicinal plants, as extracts from the leaves contain ingredients that improve memory, increase blood circulation, and benefit those suffering from Alzheimer’s disease [2,3,4]. The leaf contains multiple compounds, such as terpene trilactones, flavonoids, and phenolic acids, and the biological effect of *G. biloba* extracts has been thought to be a synergistic action of these compounds. Ginkgolides and bilobalide are unique components found only in *G. biloba* and are thought to contribute to its neuroprotective and vasotropic effects [5,6,7].

Furthermore, flavonoids, such as derivatives of (−) epicatechin, (+) catechin, apigenin, luteolin, quercetin, kaempferol, and *iso*-rhamnetin, have been found in *G. biloba* leaves [8,9]. The leaves of *G. biloba* contain numerous phenolic acids, such as caffeic acid, *p*-coumaric acid, ferulic acid, protocatechuic acid, *p*-hydroxybenzoic acid, vanillic acid, and chlorogenic acid [10,11]. The composition of terpene trilactones in *G. biloba* leaves has been shown to be dependent on the harvest date, growing location [12,13,14], and certain stresses [15,16,17,18]. Inoue et al. [19] reported that contents of ginkgolides A, B, and C in green leaves collected in August were much higher than those in fallen yellow leaves collected in November. These results raise the possibility that the senescence process, together with tissue differences (leaf blades and petioles) in leaves, affects the endogenous levels and composition of these terpene trilactones, as well as flavonoids and phenolic acids in *G. biloba* leaves.

Phytohormones play a crucial role in the development process as well as in the integration of environmental signals to various physiological processes in plants. Among them, jasmonates (methyl jasmonate (JA-Me), jasmonic acid (JA), and their related compounds (JAs)), of which JA and JA-Me were first isolated from wormwood (*Artemisia absinthium*) and *Cleyera ochnacea* as senescence-promoting substances [20,21], are essential for the regulation of senescence in plants [22,23,24,25,26,27,28]. The role of JA-Me in stress in plants is well known [29]. JAs have also been demonstrated to be involved in triggering various biochemical and physiological processes in plants [22,23,24,30,31,32,33]. For example, they play key roles in the metabolism pathway of various secondary metabolites [34,35], lignins [36,37], and phenolic acids [38,39]. However, there is little information on the effect of JAs on other secondary metabolites in *G. biloba* leaves.

Since ginkgolides and bilobalide are difficult to synthesize chemically due to their complex chemical structures and their contents in *G. biloba* leaves are low, developing methods and/or treatments to increase their biosynthesis and accumulation possesses an important medical value. In our previous study, it was shown that JA-Me applied in lanolin paste to the abaxial side of the leaves of *G. biloba* clearly induces leaf senescence indicated as chlorophyll degradation but applied to the adaxial side does not [22]. However, the effect of such application of JA-Me on important secondary metabolites in relation to leaf senescence in *Ginkgo biloba* leaves has not been demonstrated.

The objectives of this study were to investigate the effects of JA-Me treatment on the adaxial and abaxial sides of leaves, as well as the effects of aging on the levels of terpene trilactones, individual flavonoids, and phenolic acids. Another important objective was to analyze these secondary metabolites separately in the leaf blade and petiole of *G. biloba*, as their content may be affected by tissue differences.

## 2. Results

**Figure 1:**
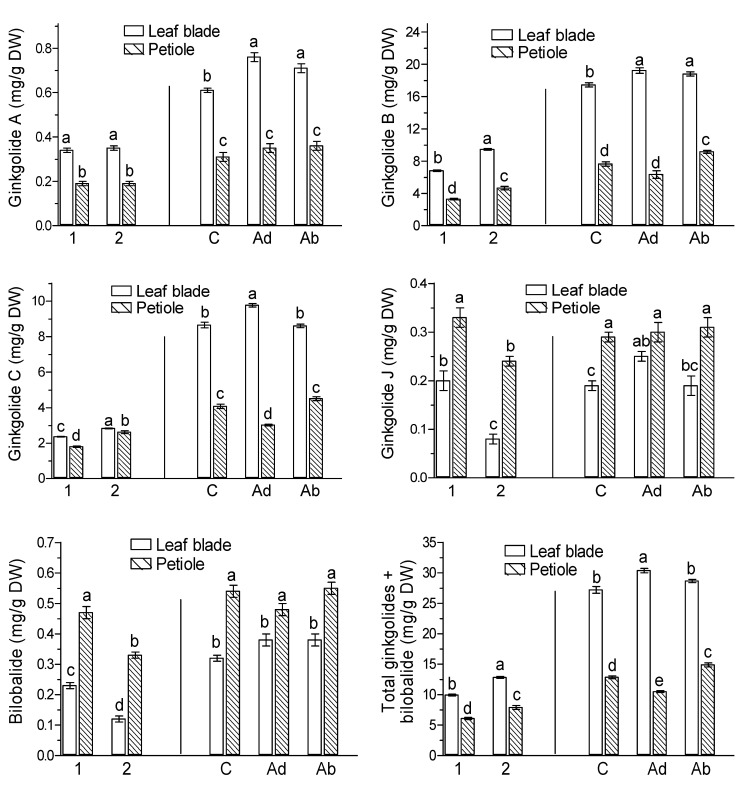
Effect of methyl jasmonate (JA-Me) on the content of ginkgolides and bilobalide in the leaf blade and petiole of *G. biloba*. Mean results ± SD followed by the same letter calculated for the natural senescence process and JA-Me treatment separately were not significantly different (*p* < 0.05) according to Tukey’s test. Description of samples: 1, initial time (9 September); 2, naturally senesced yellow leaves (20 October); C, control (lanolin); Ad, JA-Me applied to the adaxial side of the leaf blade; Ab, JA-Me applied to the abaxial side of the leaf blade. C, Ad, and Ab samples were collected for analysis on 30 September.

The green leaves of *G. biloba* collected on 9 September and naturally senesced yellow leaves collected on 20 October were found to contain specific terpene trilactones, called ginkgolides A, B, C, and J and bilobalide. There were clear differences in the contents of trilactones in the leaf blades and petioles between green leaves harvested on the day the experiment was set up (Figure 1, marked as 1) and naturally senesced yellow leaves harvested on 20 October (Figure 1, marked as 2). Among the trilactones, ginkgolides B and C were quantitatively dominant (Figure 1, marked as 1 and 2). The levels of ginkgolides A, B, and C were higher in the leaf blades than in the petioles, whereas the contents of ginkgolide J and bilobalide were about twice as high in the petioles as in the leaf blades, which may suggest that the rate of trilactone accumulation depends on the leaf tissues.

The content of ginkgolides B and C in the leaf blades was significantly higher in naturally senesced yellow leaves harvested on 20 October than in green leaves collected on 9 September (Figure 1, marked as 1), while the levels of ginkgolide J and bilobalide were slightly lower in naturally senesced yellow leaves. The same effect of natural senescence on terpene trilactones was observed in *G. biloba* petioles. This suggests that endogenous levels of ginkgolides and bilobalide are influenced by natural leaf senescence processes, with senescence positively regulating ginkgolide B and C levels.

JA-Me applied to the adaxial (Ad) and the abaxial (Ab) side of leaf blades slightly increased the endogenous levels of ginkgolides A and B in leaf blades, the promotive effect of JA-Me being ca. 16–25% and ca. 10% in ginkgolides A and B, respectively (Figure 1, Ad and Ab). However, JA-Me applied to the Ad side slightly decreased endogenous levels of ginkgolides B and C in petioles compared to the control tissue of this organ. The application sides of JA-Me, the abaxial or the adaxial, did not affect the endogenous levels of ginkgolide J in both leaf blade and petiole tissues, whereas the contents of ginkgolide J appeared to be higher in petioles than in leaf blades. In contrast, irrespective of the side of JA-Me application to the leaf blade, JA-Me slightly increased the levels of ginkgolides A and B as compared to the control, whereas JA-Me applied to the Ad side resulted in the slightly higher accumulation of ginkgolide C in leaf blades than that applied to the Ab side. JA-Me applied to the Ad side decreased the content of ginkgolides B and C in petioles compared to the control tissue of this organ.

**Figure 2:**
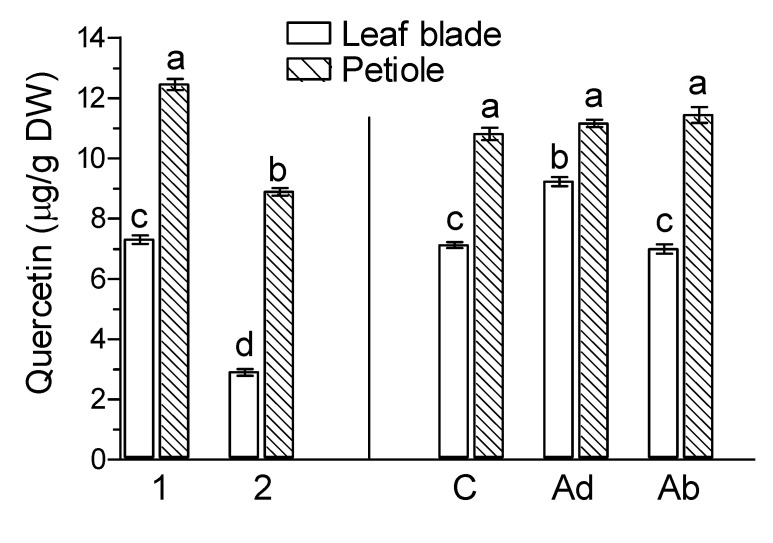

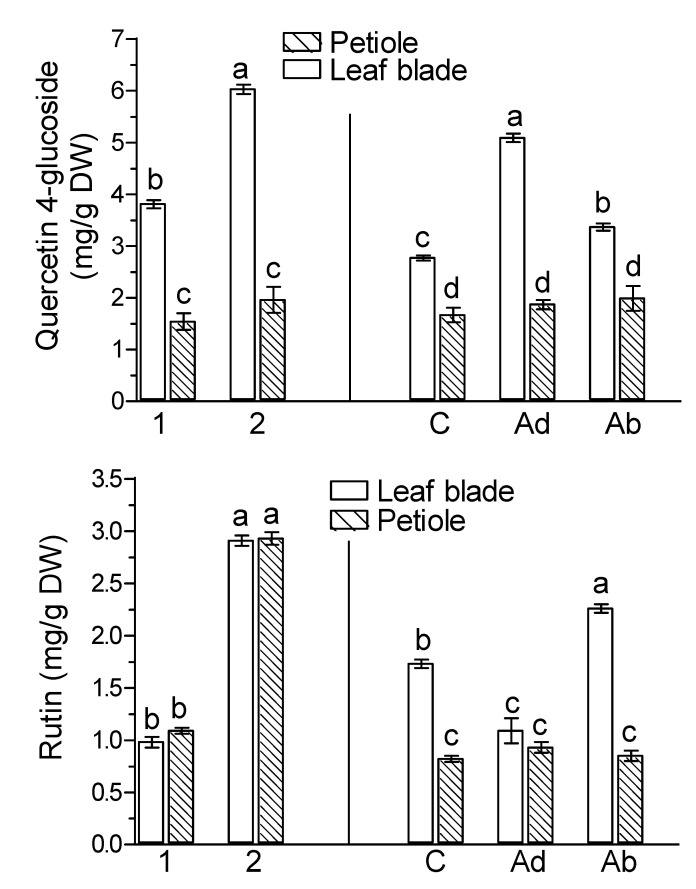
Effect of methyl jasmonate (JA-Me) on the content of flavonols in the leaf blade and petiole of *G. biloba*. Mean results ± SD followed by the same letter calculated for the natural senescence process and JA-Me treatment separately were not significantly different (*p* < 0.05) according to Tukey’s test. Description of samples: 1, initial time (9 September); 2, naturally senesced yellow leaves (20 October); C, control (lanolin); Ad, JA-Me applied to the adaxial side of the leaf blade; Ab, JA-Me applied to the abaxial side of the leaf blade. C, Ad, and Ab samples were collected for analysis on 30 September.

**Figure 3:**
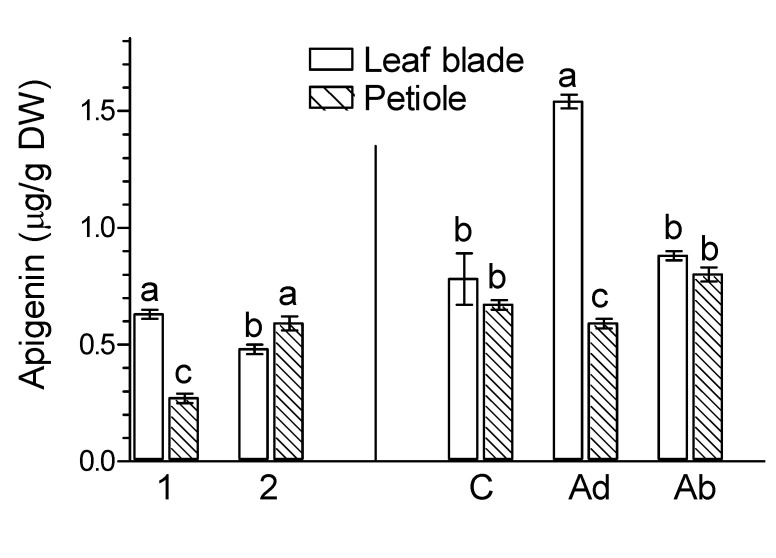

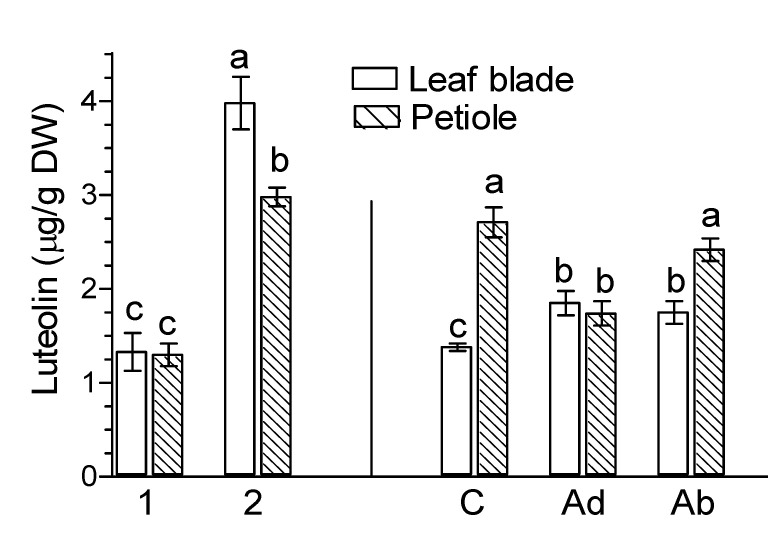
Effect of methyl jasmonate (JA-Me) on the content of flavones in the leaf blade and petiole of *G. biloba*. Mean results ± SD followed by the same letter calculated for the natural senescence process and JA-Me treatment separately were not significantly different (*p* < 0.05) according to Tukey’s test. Description of samples: 1, initial time (9 September); 2, naturally senesced yellow leaves (20 October); C, control (lanolin); Ad, JA-Me applied to the adaxial side of the leaf blade; Ab, JA-Me applied to the abaxial side of the leaf blade. C, Ad, and Ab samples were collected for analysis on 30 September.

**Figure 4:**
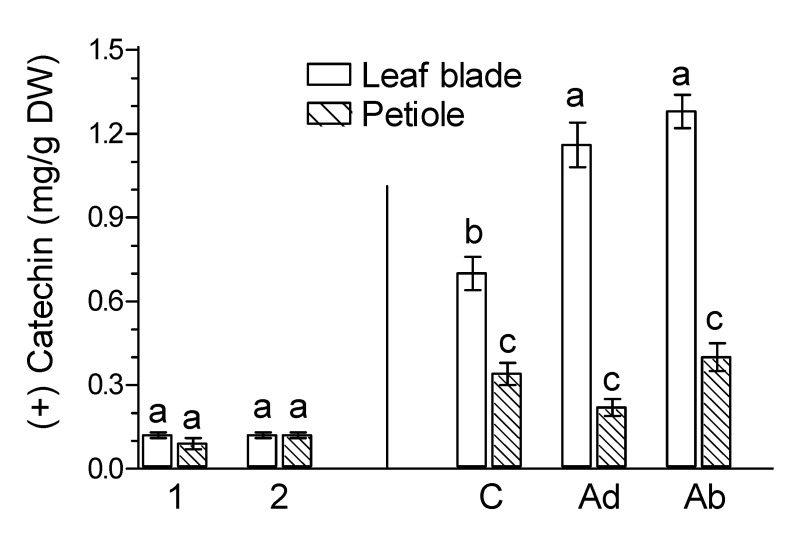

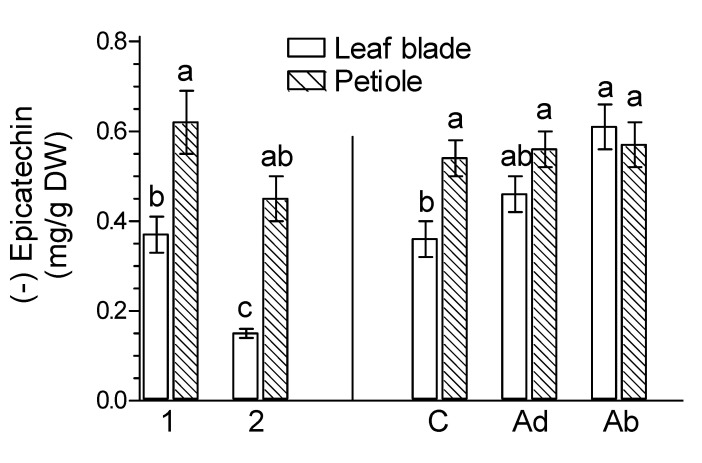
Effect of methyl jasmonate (JA-Me) on the content of (+) catechin and (−) epicatechin in the leaf blade and petiole of *G. biloba*. Mean results ± SD followed by the same letter calculated for the natural senescence process and JA-Me treatment separately were not significantly different (*p* < 0.05) according to Tukey’s test. Description of samples: 1, initial time (9 September); 2, naturally senesced yellow leaves (20 October); C, control (lanolin); Ad, JA-Me applied to the adaxial side of the leaf blade; Ab, JA-Me applied to the abaxial side of the leaf blade. C, Ad, and Ab samples were collected for analysis on 30 September.

In leaf tissues of *G. biloba*, flavonols, such as quercetin and its glycosides, such as quercetin-4-glucoside and rutin (quercetin-3-rhamnosyl glucoside), were found, in addition the flavan-3-ols (−) epicatechin and (+) catechin as well as the flavones apigenin and luteolin (Figure 2, Figure 3 and Figure 4). There were substantial differences in the levels of the particular flavonoids in examined leaf tissues between green leaves harvested on 9 September when the experiment was set up (Figure 2, marked as 1) and naturally senesced yellow leaves harvested on 20 October (Figure 2, marked as 2). The contents of flavonoids in the leaf blades and petioles of *G. biloba* were almost the same (Figure 2). The levels of quercetin 4-glucoside and rutin in the leaf blades and petioles of naturally senesced yellow leaves collected on 20 October (Figure 2, marked as 2) were significantly higher than those of green leaves collected on 9 September (Figure 2, marked as 1), but the level of quercetin in leaf blades and petioles was higher in green leaves.

The levels of apigenin in the leaf blades and petioles of ginkgo leaves varied slightly during natural senescence (Figure 3). However, in naturally senesced yellow leaf organs (Figure 3, marked as 2), luteolin levels were about twice as high as in green leaves (Figure 3, marked as 1). The level of (+) catechin in the leaf blades and petioles of *G. biloba* was almost the same as in green leaves (Figure 4, marked as 1) and naturally senesced yellow leaves (Figure 4, marked as 2). In contrast, the levels of (−) epicatechin in the leaf blades and petioles of naturally senesced yellow leaves (Figure 4, marked as 2) were much lower than those in green leaves (Figure 4, marked as 1). This suggests that endogenous levels of flavonoids are affected differently by natural leaf senescence processes.

Irrespective of the application side, JA-Me did not affect the endogenous levels of quercetin in petioles, but JA-Me applied to the Ad side of leaf blades increased its level in the leaf blades (Figure 2). In contrast, JA-Me applied to the Ad side increased endogenous levels of quercetin 4-glucoside in the leaf blades more compared to JA-Me applied to the Ab side. For rutin, JA-Me applied to the Ab side increased its level relative to control leaf blades, whereas JA-Me applied to the Ad side decreased its content.

The levels of the flavones apigenin and luteolin in *Ginkgo* leaf organs were low (Figure 3). JA-Me applied to the Ad and Ab sides increased the levels of both flavones in leaf blades. However, the increase in the apigenin content by JA-Me applied to the Ab side was significant. In petioles, the application of JA-Me to the Ab side had some effect but tended to decrease luteolin levels.

As flavan-3-ols, the levels of (+) catechin in control leaves was much higher in leaf blades than in petioles. In contrast, the levels of (−) epicatechin were higher in petioles than in leaf blades (Figure 4). The application of JA-Me to the Ad and Ab sides almost doubled the levels of (+) catechin in leaf blades. In the case of (−) epicatechin, only JA-Me applied to the Ab side caused this phenomenon. Both methods of treatment of *G. biloba* leaves did not affect the contents of (+) catechin and (−) epicatechin in the petiole.

**Figure 5:**
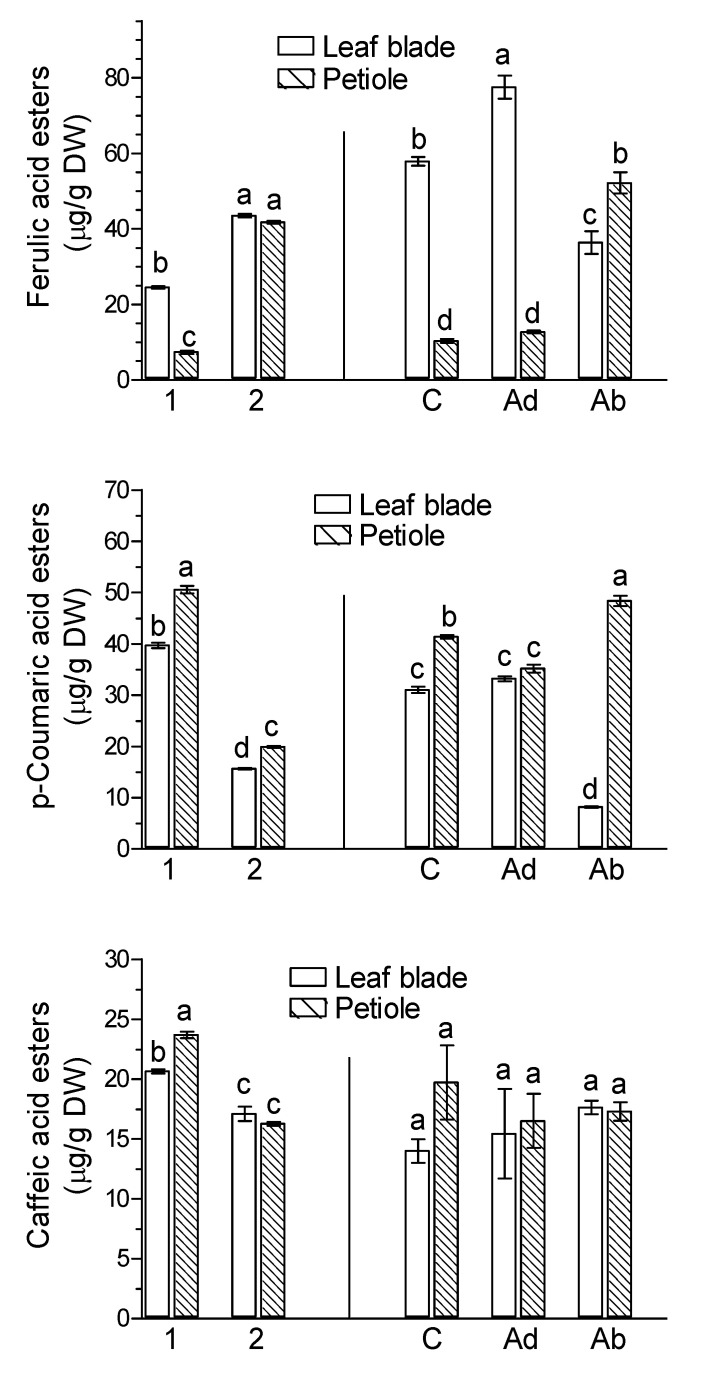

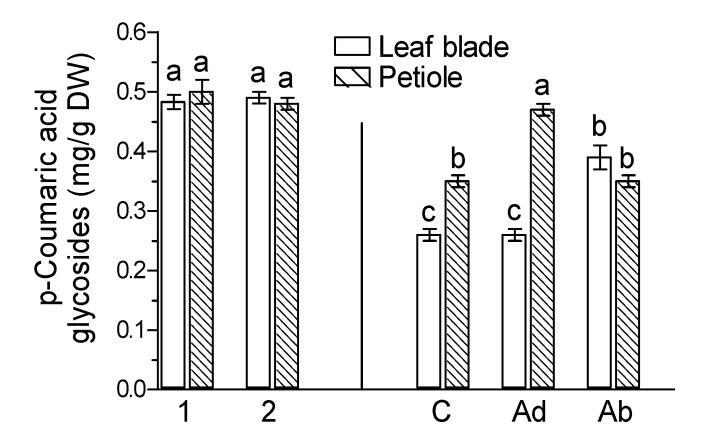
Effect of methyl jasmonate (JA-Me) on the content of phenolic acids in the leaf blade and petiole of *G. biloba*. Mean results ± SD followed by the same letter calculated for the natural senescence process and JA-Me treatment separately were not significantly different (*p* < 0.05) according to Tukey’s test. Description of samples: 1, initial time (9 September); 2, naturally senesced yellow leaves (20 October); C, control (lanolin); Ad, JA-Me applied to the adaxial side of the leaf blade; Ab, JA-Me applied to the abaxial side of the leaf blade. C, Ad, and Ab samples were collected for analysis on 30 September.

Among the phenolic acids, ester derivatives of caffeic acid and ferulic acid as well as *p*-coumaric acid esters and glycosides were found. The level of *p*-coumaric acid glycosides in leaf blades and petioles was about 10 times higher than their ester form. However, no free forms of phenolic acids were found (Figure 5). There were clear differences in the contents of individual phenolic acids in leaf blades and petioles between green leaves harvested on the day of the experiment (Figure 5, marked as 1) and in naturally senesced yellow leaves (Figure 5, marked as 2).

The levels of ferulic acid esters and *p*-coumaric acid esters were much higher in the petioles than in the leaf blades, whereas the levels of caffeic acid esters and *p*-coumaric acid glycosides were almost the same between leaf blades and petioles (Figure 5). The contents of ferulic acid esters in both leaf blades and petioles were much higher in naturally senesced yellow leaves (Figure 5, marked as 2) than in green leaves (Figure 5, marked as 1). Contrarily, the contents of ester derivatives of caffeic and *p*-coumaric acids in both leaf blades and petioles were much lower in naturally senesced yellow leaves (Figure 5, marked as 2) than in green leaves (Figure 5, marked as 1). The level of *p*-coumaric acid glycosides in both petioles and leaf blades did not differ between green (Figure 5, marked as 1) and naturally senesced yellow leaves (Figure 5, marked as 2). These results suggest that the metabolism of phenolic acids is affected as is the metabolism of flavonoids.

The application JA-Me to the Ad side increased the levels of ferulic acid in the leaf blades of *Ginkgo* leaves, whereas that of JA-Me applied to the Ad side markedly decreased it compared with the control (Figure 5). The application of JA-Me to the Ab side substantially decreased the content of *p*-coumaric acid esters but increased that of *p*-coumaric acid glycosides in the leaf blades of *Ginkgo* leaves. In petioles, the opposite effect of JA-Me on *p*-coumaric acid esters and *p*-coumaric acid glycosides was observed. In the case of caffeic acid esters, JA-Me applied to the Ad side did not affect their levels in both leaf blades and petioles of *G. biloba*.

## 3. Discussion

Tissues of *Ginkgo biloba* leaves contain pharmaceutically valuable ginkgolides and bilobalide, flavonoids, and phenolic acids. For scientific and pharmacological reasons, it is important to develop methods to increase their biosynthesis and accumulation. As previously reported, JA-Me applied to the abaxial (lower, Ab) side of the leaf blade center significantly induced leaf yellowing or aging and accelerated chlorophyll degradation three weeks after application on 9 September, while control leaves remained green [22]. In contrast, no yellowing was observed in leaves treated with JA-Me on the adaxial (Ad) side of the leaf blade center, and there was almost no difference in the leaf color compared to control leaves [22].

To the best of our knowledge, whole leaves are used to determine the content of terpene trilactones in *G. biloba* [13,14,16,17,18], but we do not fully know the details of these analyses. However, the results of the present study showed that the total content of terpene trilactones is much higher in leaf blades than in petioles, while the contents of flavonoids and phenolic acids in leaf blades are not different from those in petioles. This useful fact indicates that mainly the leaf blades of *G. biloba* should be used for the production of valuable commercial and medicinal compounds.

It has been shown that the composition and content of terpene trilactones (TTLs) in *G. biloba* leaves depends on the harvest time and cultivation site [17,18], as well as on certain stresses [19]. The levels of ginkgolides A, B, and C in green leaves collected in August were much higher than those in fallen yellow leaves collected in November [17]. Therefore, the metabolism of TTLs in leaves naturally senesced on the tree may be different from that in leaves senesced after abscising. However, as shown in Figure 1, the levels of total TTLs in naturally senesced yellow leaves collected on 20 October (2) was higher than in green leaves collected on 9 September (1). TTLs levels were highest after the 11-day treatment (September 20) (Figure 1), indicating that they increase during the growing and leaf maturation period and then decrease during senescence. Similar seasonal changes in these compounds have been found in leaves of young seedlings from the female *Ginkgo* tree [40,41,42]. Thus, mature leaves are recommended as appropriate materials to collect the *Ginkgo* leaf extract for pharmaceutical use.

There are no data in the available literature on the effect of the JA-Me application site on *G. biloba* leaves on the composition and content of the metabolites present in leaf organs. Therefore, whether the leaf blade and petiole respond similarly to JA-Me treatment seems important. As shown in Figure 1, the current study demonstrated that JA-Me applied to the adaxial (Ad) or abaxial (Ab) side increased the level of TTLs in leaf blades, especially ginkgolides B and A, as well as total TTLs. The stimulatory effect of JA-Me on TTL accumulation was also found in the petiole of *G. biloba*. Regardless of the mode of application, JA-Me had little effect on the relative composition of terpene trilactones, although their composition in petioles was slightly different from that in leaf blades. This indicates that the application of JA-Me to the green leaves of *G. biloba* may have practical significance.

Results of our previous study have shown that JA-Me applied to the abaxial side of leaves significantly stimulates leaf senescence and significantly affects the contents of endogenous JA-Me and ABA in both leaf blades and petioles [22]. In addition, it was shown that in *G. biloba*, the stomata and numerous cuticular folds are present only on the abaxial side of the leaf. Furthermore, the cells of the adaxial epidermis appear stretched rather than wrinkled as in the abaxial epidermis [43]. In contrast, JA-Me in lanolin paste applied to the Ad and Ab sides substantially increased the TTLs in leaf blades (Figure 1). The levels were almost the same as those in senescent leaves induced by the application of JA-Me to the Ab side and still green leaves treated with JA-Me on the Ad side (Figure 1). This may indicate that senescence processes and changes in the levels of some phytohormones after JA-Me treatment precede the biosynthesis reaction of TTLs. Furthermore, the different effects of JA-Me on TTLs in leaf blades and petioles are presumably due to the rate of JA-Me transport and/or accumulation.

To date, studies of the effects of JA-Me on TTL biosynthesis have been conducted using tissue culture or by spraying young trees or seedlings of *G. biloba*. In general, tissue cultures are a convenient method to study the effects of various factors on the biosynthesis and accumulation of secondary metabolites [44]. However, the contents of TTLs in tissue cultures are much lower compared with the levels in leaves growing on *G. biloba* trees. Thus, JA-Me and salicylic acid have often been used to increase the biosynthesis of essential secondary metabolites [45,46]. These studies showed that the use of JA-Me in *G. biloba* cell culture increases TTLs but at the same time slightly decreases cell growth [47]. The applied JA-Me strongly increased the production of these metabolites in cells and stimulated their release into the culture medium. Sukito and Tachibana [46] showed that JA-Me elicitation in immobilized *G. biloba* cell cultures increases the production of TTLs, and synergism between JA-Me and salicylic acid was found. In our study, JA-Me increased the content of ginkgolides A, B, and C but not bilobalide (Figure 1). Differences in the effects of JA-Me on TTLs may be due to distinct experimental conditions.

The key enzyme involved in the terpene trilactone biosynthesis pathway is 3-hydroxy-3-methylglutaryl coenzyme A reductase (HMGR). According to Liao et al. [47] and Rao et al. [48], the TTL content in *G. biloba* is regulated by factors such as JA-Me, ABA, SA, ethylene, darkness, and cold. These factors induce the expression of GbHMGR2 and GbHMGR3 genes, which can directly participate through the mevalonic acid (MVA) pathway in TTL biosynthesis. Spraying *G. biloba* seedlings with JA-Me, as well as with salicylic acid and ethephon solutions, significantly increased the levels of TTLs, and the accumulation was positively correlated with the expression level of key genes. In contrast, ABA use had no effect on the content of TTLs [44,45,46].

In a study by Zhang et al. [42], the effect of spraying 10-year-old *G. biloba* trees with gibberellic acid (GA) on 25 June on the contents of indolyl-3-acetic acid (IAA), ABA, GA, and ginkgolides in leaves was investigated from 10 July to 25 October. It was found that the highest accumulation of TTLs was from late August to late September. The application of GA significantly increased the TTL content, and the endogenous GA levels reduced in the leaves, while the IAA and ABA contents varied. In contrast, Lin et al. [15] found, by studying the dynamics of changes in the contents of total TTLs and flavonoids, that October is the optimal time to harvest *G. biloba* leaves. An analysis of gene expression related to leaf senescence in *G. biloba* showed that the expression levels of most ABA- and JA-related genes increases, whereas genes related to the cytoskeleton, photosynthesis, and antioxidant biosynthesis decreases from the green leaf stage to the yellow leaf stage [49]. Furthermore, Ye et al. [50] showed that salicylic acid application can upregulate TTLs, in whose biosynthesis 30 genes may be involved.

JA-Me applied to the adaxial (Ad) side of the leaves increased the contents of quercetin, quercetin 4-glucoside, apigenin, and catechin in the leaf blades of *G. biloba*. However, JA-Me applied to the abaxial (Ab) side of the leaves had little effect on flavonoid contents. This may indicate that senescence processes after JA-Me treatment somehow inhibit the flavonoid biosynthesis reaction. The effect of JA-Me on the content of flavonoids in petioles was lower than in leaf blades, suggesting that the different effects are presumably due to the rate of JA-Me transport and/or accumulation, as suggested above.

Ferulic acid (4-hydroxy-3-methoxy-cinnamic acid) and *p*-coumaric acid (4-hydroxy-cinnamic acid) are common constituents in the cell walls of plants [51,52,53,54]. Ferulic acid plays a key role in providing cell wall rigidity and has some antioxidant activity [55]. In the primary cell walls of higher plants, ferulic and *p*-coumaric acids are linked by ester bonds to polysaccharides, which affects the extensibility and biodegradability of the cell wall [56,57,58]. It is possible that the decrease in ferulic and *p*-coumaric acid esters in the leaf blade and the increase in their content in the petiole after application of JA-Me to the Ab side observed in our study is related to induced leaf senescence and finally to accelerated leaf death through changes in cell wall extensibility and biodegradability. A certain confirmation of this supposition is provided by our recently published results of a great increase in ABA content under the influence of the application of JA-Me to the Ab side, which directly affects the senescence process and the formation of a cut-off layer in the petiole [22]. Hydroxycinnamic acids appear to be associated with the plant abiotic stress response. It was also found that osmotic stress suppresses cell wall stiffening and the increase in cell-wall-bound ferulic and diferulic acids in wheat coleoptiles and *Avena* coleoptiles [59,60]. Hura et al. [61] suggested that an increase in the content of cell-wall-bound phenolic acids improves the tightness of the cell wall and protects cells against water loss in the dehydrated leaves of triticale.

It is known that jasmonate biosynthesis in plants is stimulated by various abiotic stresses and biotic stresses, such as pathogen infections and insect wounds. [27,29,30,31,32]. The effects of JA-Me on chlorophyll degradation and changes in the phytohormone status appear to be much faster than on the metabolism of secondary compounds in *G. biloba* leaves. This raises the question of why JA-Me alters phenolic acids when applied to the Ab side but has limited effects on TTLs. It is likely that both the complex chemical structure and the biosynthetic pathway of TTLs result in a much slower response to JA-Me than to phenolic acids. The differences between the phenolic composition of *G. biloba* leaves in our study and previously published ones may also be due to the fact that several environmental factors modify the accumulation of secondary metabolites in plants [15,62].

Earlier, Sati and Pandey [63] found that tree age is an important factor affecting the accumulation of secondary metabolites in *G. biloba* leaves. Another important factor is genetic differences between plants [64]. Added to this is the influence of the method used to isolate phenolic compounds and the technique used to analyze them [65,66].

## 4. Materials and Methods

### 4.1. Plant Materials

A 12-year-old *G. biloba* tree growing in Skierniewice, Poland (51°58′29.5″ N 20°09′05.1″ E), was used in this study. The gender of *G. biloba* trees used in this study has not been clarified yet [40]. However, Jin et al. [41] reported that male and female *G. biloba* have similar responses to light in several photosynthetic traits. All the treatments were carried out on different branches of one tree, and green leaves were collected from the same tree as controls. More than 20 leaves from different branches were used for each treatment. The green leaves were treated with methyl jasmonate (JA-Me) at a concentration of 0.5% (*w*/*w*) in lanolin paste containing 30% water (*w*/*w*), as previously described [22]. It was applied as a 2–3-mm-wide strip in the middle part of the leaf on the adaxial (upper, Ad) and/or abaxial (lower, Ab) side, and lanolin with 30% water content was applied in the same way as a control. The treatments with JA-Me were initiated on 9 September 2017. Three weeks after treatment, JA-Me-treated leaves and control ones were harvested, freeze-dried, and subjected to analyses of ginkgolides and bilobalide, as well as flavonoids and phenolic acids (free, esters, and glycosides separately). For comparison with JA-Me treatment, naturally senesced yellow leaves were collected on 20 October and subjected to the analysis.

### 4.2. Analyses of Terpenoic Trilactones (Gingkolides, Bilobalide), Flavonoids, and Phenolic Acids

The secondary metabolite mentioned above was determined separately in the leaf blade and petiole of *G. biloba* using HPLC-MS/MS according to the method of Płatosz et al. [67]. Briefly, crude extracts were obtained from freeze-dried plant samples by stirring them overnight at 10 °C using a ThermoMixer (Benchmark Scientific, Saryeville, NJ, USA) with a mixture of methanol, water and formic acid 80:19.9:0.1 (*v*/*v*/*v*). The extraction was repeated five times, and the obtained crude extracts were collected. Using these crude extracts, gingkolides A, B, C, and J and bilobalide were analyzed using HPLC-MS/MS. In contrast, free forms of flavonoids and phenolic acids were fractionated with diethyl ether after adjusting the initial extract to pH 2 with 6 N HCl. After free forms were fractionated into diethyl ether, ester forms of these compounds present in the residues were hydrolyzed in a nitrogen atmosphere for 4 h at room temperature with 4 M NaOH. Subsequently, glycosides present in the residues were hydrolyzed with 6 M HCl for 1 h at 100 °C. After each step, the released free forms were isolated with diethyl ether after adjusting the mixture to pH 2. The obtained diethyl ether fractions were evaporated to dryness under a stream of nitrogen at 35 °C. The obtained compounds were dissolved in 80% (*v*/*v*) methanol, centrifuged, and subjected to HPLC-MS/MS analysis.

An HPLC system equipped with an HALO C18 column (2.7 μm particles, 0.5 × 50 mm^2^; Eksigent, Vaughan, ON, Canada) at 45 °C at a flow rate of 15 μL/min was used. As the elution solvents, a gradient of solvent A (water:formic acid 99.05:0.95 *v*/*v*) and solvent B (acetonitrile:formic acid 99.05:0.95 *v*/*v*) were used as follows: 5% solvent B for 0.1 min, 5–90% solvent B for 1.9 min, 90% B for 0.5 min, 90–5% B for 0.2 min, and 5% B for 0.3 min. For HPLC-MS/MS analysis, a QTRAP 5500 ion trap mass spectrometer (AB SCIEX, Vaughan, ON, Canada) was applied. Optimal ESI-MS/MS conditions including nitrogen curtain gas (25 L/min), collision gas (9 L/min), ion spray source voltage (–4500 V), temperature (350 °C), nebulizer gas (35 L/min), and turbo gas (30 L/min) were applied. Qualitative and quantitative analyses were conducted in negative mode by multiple reaction monitoring (MRM) of selected ions in the first quadrupole and the third quadrupole (Supplementary Table S1).

The standards of ginkgolides A, B, C, and J, bilobalide, flavonoids, and phenolic acids were purchased from Sigma Chemical Co. (St. Louis, MO, USA). The external standards (0.01–0.50 µg/mL) showed linear calibration curves with a high correlation coefficient (0.997–0.999).

### 4.3. Statistical Analysis

One-way analysis of variance (ANOVA) and Tukey’s post hoc test were used to check the significance of differences in the petiole and leaf blade of *G. biloba*. Comparisons were made between natural senescence and JA-Me treatment separately. Calculations were performed using Statistica 12PL software (StatSoft, Tulsa, OK, USA). Means ± SD followed by the same letter were considered statistically insignificant at *p* > 0.05 (post hoc Tukey’s test).

## 5. Summary

Terpene trilactones (TTLs), flavonoids, and phenolic acids in *Gikgo biloba* leaves show dynamic changes during leaf maturation and senescence. Application of JA-Me to green leaves, regardless of the application site, adaxial or abaxial, significantly increases the levels of TTLs. The applied JA-Me also affects the levels of some flavonoids and phenolic acids. Quantitative and qualitative studies of TTLs, flavonoids, and phenolic acids of *G. biloba* leaves may provide information useful for *Ginkgo* cultivation, leaf quality improvement, and chemical regulation of beneficial components.

It seems that the effect of JA-Me on chlorophyll degradation and changes in the phytohormone status is much faster than on the metabolism of secondary compounds in *G. biloba* leaves. However, judging from the results of the present study, JA-Me-mediated chemical regulation is somewhat useful for regulating endogenous secondary metabolites in *G. biloba*. In the present study, it was found that application of JA-Me to the adaxial and abaxial sides of *G. biloba* leaves generally increases the content of individual TTLs. Effects of JA-Me on the contents of some phenolic acids and flavonoids were also found. In conclusion, it can be stated that the application of JA-Me influences the processes of leaf senescence and modifies the accumulation of ginkogolides, flavonoids, and phenolic acids. These effects were also found to be different in leaf blades and petioles.

## Data Availability

The data presented in this study are available in this article.

## Supplementary Materials

The following are available online, Table S1: Data on the analysis of terpene trilactones (TTLs), phenolic acids, and flavonoids in *Gingko biloba* leaves using HPLC-MS/MS.

## Sample Availability

Samples of the compounds are not available from the authors.
