# Supplementary material for: Effect of Methyl Jasmonate on the Terpene Trilactones, Flavonoids, and Phenolic Acids in Ginkgo biloba L. Leaves: Relevance to Leaf Senescence
Source: Molecules. 2021 Aug 2;26(15):4682. doi: 10.3390/molecules26154682 (PMC8347123; doi:10.3390/molecules26154682)
Supplement: Supplementary file 1 [file molecules-26-04682-s001.zip › molecules-1316539-supplementary.pdf]

Supplementary Table 1. Data on the analysis of terpene trilactones (TTLs), phenolic acids and flavonoids in *Gingko biloba* leaves using HPLC-MS/MS

Analyzed chemical	Retention time (min)	MS/MS (m/z)
Ginkgolide A	1.83	407/351
Ginkgolide B	1.83	423/367
Ginkgolide C	1.60	439/353
Ginkgolide J	1.67	425/163
Bilobalide	1.67	325/163
Rutin	1.44	609/301
Quercetin 4-glucoside	1.51	463/301
(+) Catechin	1.38	289/245
(-) Epicatechin	1.30	289/245
Quercetin	1.83	301/151
Apigenin	1.92	269/151
Luteolin	1.60	285/133
Ferulic acid	1.35	193/134
p-Coumaric acid	1.53	163/119
Caffeic acid	1.57	179/135
